# Specialist palliative care until the very end of life - reports of family caregivers and the multiprofessional team

**DOI:** 10.1186/s12904-023-01266-6

**Published:** 2023-10-10

**Authors:** Anneke Ullrich, Sven Goldbach, Wiebke Hollburg, Bettina Wagener, Annette Rommel, Marten Müller, Denise Kirsch, Katrin Kopplin-Foertsch, Holger Schulz, Carsten Bokemeyer, Karin Oechsle

**Affiliations:** 1 Palliative Care Unit, Department of Oncology, Hematology and BMT, University Medical Center Eppendorf, Hamburg, Germany; 2 Specialist outpatient palliative care team ‘PalliativPartner Hamburg GbR’, Hamburg, Germany; 3 Specialist outpatient palliative care team ‘Das Palliativteam’, Hamburg, Germany; 4 Palliative care ward, Asklepios Hospital Rissen, Hamburg, Germany; 5 Specialist outpatient palliative care team ‘PCT Hamburg-West’, Hamburg, Germany; 6 Palliative care ward, Agaplesion Diakonie-Hospital, Hamburg, Germany; 7 Department of Medical Psychology, University Medical Center Eppendorf, Hamburg, Germany

**Keywords:** Specialist palliative care, End-of-life care, Multiprofessional team, Family caregiver, Palliative Care Outcome Scale

## Abstract

**Background:**

Specialist palliative care (SPC) includes care for incurably ill patients and their family caregivers at home or on a palliative care ward until the very end of life. However, in the last days of life, patients can rarely express their needs and little is known about SPC outcomes as reported by multiprofessional SPC teams and family caregivers.

**Methods:**

Using the Palliative Care Outcome Scale (POS; Score 0–40), proxy assessments of SPC outcomes in the patient’s last 3 days of life were performed by SPC teams and primary family caregivers of three home care and three inpatient services. Additional questions were asked about problems solved ‘particularly well’ or ‘inadequately’ (last 7 days), which were content analyzed and quantified.

**Results:**

Proxy assessments by SPC teams were available in 142 patients (of whom 51% had died at home). Family caregiver assessments exist for a subgroup of 60 of these patients. SPC teams (POS total score: mean 13.8, SD 6.3) reported SPC outcomes slightly better than family caregivers (mean 16.7, SD 6.8). The POS items consistently rated as least affected (= 0) by both, SPC teams and family caregivers, were ‘not wasted time’ (team 99%/family caregivers 87%), ‘information’ (84%/47%) and ‘support’ (53%/31%). Items rated as most affected (= 4) were ‘patient anxiety’ (31%/51%), ‘life not worthwhile’ (26%/35%) and ‘no self-worth’ (19%/30%). Both groups indicated more problems solved ‘particularly well’ than ‘inadequately’; the latter concerned mainly clinically well-known challenges during end-of-life care and family caregiver care.

**Conclusions:**

This study shows the range and type of symptoms and other concerns reported in the patient’s last days. Starting points for further improvements in family caregiver care and psychosocial and spiritual issues were identified.

**Supplementary Information:**

The online version contains supplementary material available at 10.1186/s12904-023-01266-6.

## Background

Palliative care aims to improve quality of life in patients suffering from incurable, progressive and life-limiting diseases and their family caregivers [1]. Although palliative care focusses mainly on the patients’ quality of life during the often-long disease trajectory, it also includes care towards the end of life including the terminal care for patients in their last days of life. In Germany, specialist palliative care (SPC) can be administered by specialized multiprofessional teams in specific care settings, either as specialist inpatient palliative care on a palliative care ward or as specialist outpatient palliative care (SAPV) at the patient’s home [2]. SAPV is also feasible in patients receiving care in inpatient hospices and nursing homes. SAPV is provided by multiprofessional home palliative care teams, comprising at least specialized physicians and nurses [3].

Patient reported outcome measures (PROMS) are considered standard in clinical practice to ensure adequate patient care during the whole course of SPC [1]. However, the last days in a patient’s life represent a specific situation, as the majority of patients is unable to report their problems and needs themselves. Thus, patient care has to be navigated based on proxy assessments by multiprofessional SPC teams and family caregivers. A longstanding, well-established standard instrument for patient and proxy assessment during SPC is the ‘Palliative Care Outcome Scale’ (POS) [4–7]; now called the ‘Integrated Palliative Care Outcome Scale’ [8]. End-of-life care has also been evaluated using specific proxy assessment tools like the ‘Quality of Dying and Death Questionnaire’ (QoDD) [9–12] or the ‘Care of the Dying Evaluation’ (CODE™) [13–15] in varying, but mainly non-specialist palliative care settings.

With regard to assessing a dying patient’s symptom burden and needs, differences of family caregivers’ assumptions compared to health care professionals’ assessment may happen: Family caregivers’ subjective experience of the patient’s situation might be influenced by their own anxiety and burden [16, 17] and therefore, they may be less objective proxies than health care professionals are. Health care professionals in turn might underestimate symptom burden or overestimate quality of life as they spend less time with the patient than family caregivers [18, 19]. This dilemma might be mirrored by the heterogeneous results of studies, which mainly, but not consistently report low levels of congruence between the ratings of family caregivers and health care professionals [19–21], especially concerning aspects of psychosocial care [21].

Therefore, the aim of our exploratory study was (1) to describe SPC outcomes in the patient’s last days of life from the perspectives of multiprofessional SPC teams and family caregivers. In addition to symptoms and problems assessed by PROM measures, we were interested in subjective accounts on problems that could be solved ‘particularly well’ or remained to be solved ‘inadequately’. Further aims were (2) to compare these two perspectives of SPC teams and family caregivers, and (3) to identify factors associated to SPC outcomes.

## Methods

### Study design and participants

The present study is part of a large prospective, longitudinal, multicenter observational protocol evaluating support needs, psychological problems and personal last wishes of patients during their inpatient and outpatient SPC in the metropolitan area of Hamburg, Germany [22, 23]. This research mainly focused on the patient perspective. To evaluate SPC outcomes until the very end of life, retrospective proxy assessments by adult, primary family caregivers (including family, relatives and friends) and multiprofessional SPC teams were included.

Within the superordinate study, patients were consecutively enrolled in six SPC services of an urban network, including three outpatient SPC services and three palliative care wards between June 2017 and July 2018. The study follow-up period ended in December 2018. Previously trained staff of the participating services recruited patients within 72 h after initiation of SPC. Incurably ill patients were eligible if they were entering in- or outpatient SPC for the first time and were at least 18 years old. Exclusion criteria included cognitive or language limitations, acute critical physical or psychological problems, and imminent death of patients. Additional details on the study design have been described elsewhere [22, 23].

According to the study-protocol, multiprofessional SPC teams completed a questionnaire including the German version of the Palliative Care Outcome Scale (POS) [4–7] within one week after the patients’ death, if the patient had died in the respective service. SPC teams were instructed that the questionnaire was to be completed by a team member who had personally cared for the patients within the final days of life. Respecting the extraordinary burden in the first weeks after a loved one’s death, family caregivers completed the same questionnaire within 6–8 weeks post-loss. The person, who had been indicated as the primary family caregiver by the patient and had consented to participate in the study, received the questionnaire together with a pre-paid envelope by mail. To comfort the recently bereaved, the study material and a personalized cover letter were sent by the treating SPC team.

The Ethics Committee of the General Medical Council of Hamburg, Germany, approved the study protocol (PV5062). Written informed consent was mandatory for all participating patients and included consent for an assessment post-bereavement by the treating SPC team and the family caregiver indicated by the patient. Additionally, these family caregivers had to sign a written informed consent form for study participation.

### Measurements

The questionnaires for SPC teams and family caregivers included closed and open-ended questions on SPC outcomes in the patient’s last days of life.

The German version of the 3-day recall Palliative Care Outcome Scale (POS) [4–7] was used for assessment of multidimensional SPC outcomes, as the validation of the later Integrated Palliative Care Outcome Scale (IPOS) – with associated German IPOS – was published in 2019 [8], thus after the data collection period (June 2017 to December 2018). The POS relates to how someone is affected by a symptom or concern. The proxy versions consist of 10 items with 5 response options scoring from 0 (= item rated as least affected) to 4 (= item rated as most affected). The total score ranges from 0 to 40 points, with lower values indicating better outcomes [6]. Additionally, the health care professional version includes a question about the patient’s performance status according to the Eastern Cooperative Oncology Group (ECOG) [24].

In the open part of the questionnaire, two additional questions were asked about problems solved ‘particularly well’ or ‘inadequately’ during the week preceding the patient’s death. These non-validated questions are used in the national German database ‘Hospice and Palliative Care Evaluation’ (HOPE) [25–28]. In this study, they were added in to the POS measure. SPC teams and family caregivers were asked: ‘Which problems could be solved particularly well during the last 7 days of the patient’s life?’ and ‘Which problems could only be solved ‘inadequately’ during the last 7 days of the patient’s life?’. As literature gives reason to believe that large answer spaces for open-ended questions are likely to improve the quality of responses [29], answer spaces of several lines were designed.

In addition, the SPC teams reported about the patient’s dying situation in terms of location at death (home, inpatient hospice, nursing home, and SPC ward), SPC setting (outpatient vs. inpatient), vigilance status in the last 3 days of the patient’s life, and whether somebody was with the patient at the moment of death.

Data on patients’ socio-demographic, disease- and care-related characteristics were retrieved from the superordinate study database.

### Statistical analysis

Descriptive data are reported in frequency and proportions for categorical variables, and means (with standard deviation) for continuous variables. We calculated mean paired differences in POS items and the POS total score at a group level (SPC teams vs. family caregivers) using paired t-tests.

To estimate the agreement and correlations of ratings of categorized POS items, weighted Cohen’s kappa (κ_*w*_) and Spearman’s rho (r_s_) were calculated. According to Landis and Koch (1977), kappa should be 0.61–0.80 in order to assume a substantial agreement [30]. According to the benchmarks of Cohen, r_s_ ≥ 0.50 represents large, r_s_ ≥ 0.30 medium, and r_s_ ≥ 0.1 small effects [31]. To reduce complexity, the 5 response options of the POS items were categorized into three groups for these analyses. Categorization was conducted according to Bausewein et al. [6]: category 1 comprises the most positive answer (0 = item rated as least affected), category 2 ratings with 1 or 2, category 3 the most negative answer (3 and 4; 4 = item rated as most affected).

Factors potentially associated with SPC outcomes were analyzed using multivariable linear regression (enter method), with the POS total score being the dependent variable. Independent variables with more than two categories were dichotomized with values of 0 and 1. Possible multicollinearity of independent variables was tested by correlational analysis (Spearman’s r), variance inflation factors (VIFs) and tolerance indices (TIs). For the standardized coefficient *ß*, effect sizes between 0.10 and 0.29 are small, effect sizes between 0.30 and 0.49 are medium, and effect sizes of 0.50 or greater are large effects [31].

Regarding problems solved ‘particularly well’ and ‘inadequately’, free-text responses of SPC teams and family caregivers were examined by inductive content analysis. Qualitative content analysis is suitable for identifying common issues mentioned in data and measuring the frequency of different categories [32, 33]. For quantifying categories, categories were coded for the presence or absence by assigning a value of 0 (no) or 1 (yes). Frequencies were calculated on person-level as percentages of persons with at least one written account referring to the respective category.

We used the STROBE statement for improving the quality of reporting observational studies [34].

## Results

Within the data collection period (June 2017 to December 2018), 193 of 425 participating patients (45%) had died in care of the six participating SPC services. Proxy assessments of SPC teams that conform to the study protocol were available in 142 of these patients (74%). Additionally, family caregiver assessments were existent in a subgroup of 60 of these patients (31%).

### Characteristics

On the time of first entering SPC, the mean age of the 142 patients was 71.3 years (range 29–94), 57% were male, about 90% presented with malignant diseases, and 51% had initially entered outpatient SPC. On average, patients died after a mean of 53.7 days (range 0-279) following initiation of SPC. The observed number of transfers between the inpatient and outpatient SPC setting (or vice versa) in the course of SPC was mean 0.6 (range 0–8) with 32% having experienced at least one of such transfers. Dying at home with outpatient SPC was observed in 51% of patients. Details are presented in Table 1.

**Table 1:** Patient characteristics, care and dying situation (N = 142)

		n	%
Age, M (SD); range		71.3 (11.2); 29–94	
Gender	Male	81	57.0
	Female	61	43.0
Disease	Gastrointestinal cancer	33	23.2
	Cancer of the respiratory tract	30	21.2
	Urogenital cancer ^a^	43	30.3
	Other malignant diseases	23	16.2
	Non-malignant diseases	13	9.2
Family status	Single	23	16.3
	Married, life partnership	79	56.0
	Divorced, widowed	39	27.7
Children	Yes	107	75.9
	No	34	24.1
Advance directive at initiation of SPC	Yes	98	69.0
	No	44	31.0
Days between initiation of SPC and death, M (SD); Range		53.7 (60.3); 0-279	
Experience of at least one transfer between in- and outpatient SPC settings	Yes	45	31.7
	No	97	68.3
Number of transfers between in- and outpatient SPC settings, M (SD); Range		0.6 (1.2); 0–8	
Location at death	At home with outpatient SPC	73	51.4
	Nursing home with outpatient SPC	12	8.5
	Inpatient hospice with outpatient SPC	6	4.2
	Palliative care ward	51	35.9
SPC setting at the time of death	Outpatient	92	64.8
	Inpatient	50	35.2
Performance status (ECOG) in the last 3 days of life	ECOG ≤ 2	8	5.7
	ECOG 3	21	14.9
	ECOG 4	112	79.4
Vigilance status in the last 3 days of life	Alert	15	10.9
	Can be awakened	37	27.0
	Comatose	79	57.7
	Unknown	6	4.4
Somebody was with the patient in the moment of death	Yes	97	70.8
	No	30	21.9
	Unknown	10	7.3

^a^ Including all cancers of the urogenital system (e.g. prostate cancer, bladder cancer etc.) and breast cancer

Abbreviations: SPC, specialist palliative care; ECOG, performance status according to the Eastern Cooperative Oncology Group

### SPC outcome in the patient’s last days of life as reported by SPC teams and family caregivers

The SPC outcome in the patient’s last three days of life (POS total score) was rated better by SPC teams with average 13.8 of 40 points (N = 142, SD 6.3, range: 0–30) compared to family caregivers with average 16.7 points (N = 60, SD 6.8, range: 3–31; data not shown). In a sub-cohort of paired SPC teams and family caregivers (N = 60), the mean POS total scores were nearly identical and differed significantly between SPC teams and family caregivers (p = .039; see Supplement File 1, Table S1).

POS items reported as least affected (rating = 0) were similar between the multiprofessional teams (N = 142) and family caregivers (N = 60), yet differed in their proportion of frequency. These were ‘not wasted time’ with 99% in SPC teams and 87% in family caregivers, ‘information’ in 84% and 47%, and ‘support’ in 53% and 31%. Items reported as most affected (= 4) by both groups were ‘patient anxiety’ with 31% in SPC teams and 51% in family caregivers, ‘life not worthwhile’ in 26% and 35%, and ‘no self-worth’ in 19% and 30%. Detailed data on the SPC teams and family caregiver ratings on the ten POS items are displayed in Table 2.

**Table 2:** Proxy assessments of SPC teams (N = 142) and family caregivers (N = 60): POS items

POS item^a^ Over the last three days of the patient’s life:			0 (% rated as least affected)	1 (%)	2 (%)	3 (%)	4 (% rated as most affected)
1	Pain	SPC Team	27.5	17.6	27.5	23.2	4.2
		Family caregivers	21.1	15.8	26.3	35.1	1.8
2	Other symptoms	SPC Team	9.9	24.1	27.0	32.6	6.4
		Family caregivers	17.5	22.8	17.5	24.6	17.5
3	Patient anxiety	SPC Team	28.3	19.6	15.9	28.3	8.0
		Family caregivers	21.1	29.8	21.1	17.5	10.5
4	Family anxiety	SPC Team	8.7	25.4	13.0	21.7	31.2
		Family caregivers	3.4	11.9	5.1	28.8	50.8
5	Information	SPC Team	83.7	14.2	0.0	0.7	1.4
		Family caregivers	47.3	29.1	14.5	5.5	3.6
6	Support	SPC Team	52.6	15.6	11.1	8.9	11.9
		Family caregivers	31.0	15.5	8.6	13.8	31.0
7	Life worthwhile	SPC Team	17.0	20.7	20.0	16.3	25.9
		Family caregivers	29.3	15.5	12.1	8.6	34.5
8	Self-worth	SPC Team	18.3	31.3	13.7	17.6	19.1
		Family caregivers	15.8	29.8	15.8	8.8	29.8
9	Wasted time	SPC Team	99.3	n.a.	0.7	n.a.	0.0
		Family caregivers	87.3	n.a.	7.3	n.a.	5.5
10	Personal affairs	SPC Team	73.0	n.a.	19.0	n.a.	7.9
		Family caregivers	66.1	n.a.	23.2	n.a.	10.7

^a^ Questions 1 to 8: range 0 (no problems) to 4 (overwhelming problems), questions 9 and 10: scores of 0, 2, or 4 (higher scores indicate more problems)

Abbreviations: POS, Palliative Care Outcome Scale; SPC, specialist palliative care; n.a., not applicable

In the matched data pairs analysis (N = 60), mean scores of POS items differed significantly between SPC teams and family caregivers for ‘family anxiety’, ‘information’, and ‘support’. For all these symptoms, family caregivers perceived poorer outcomes than SPC teams (p = .001 to p = .010; see Supplement File 1, Table S1).

### Agreement and correlation between family caregiver and team assessments

Table 3 presents Cohen’s weighted kappa and Spearman’s rho for correlation between paired SPC teams and family caregivers proxy assessments (N = 60) of the categorized POS items. In this sub-cohort, prevalence of some items differed clinically relevantly (defined as > 10% difference) between SPC teams and family caregivers. Concerning items rated as most affected (rating = 3–4), these were: ‘family anxiety’ (SPC teams: 53%, family caregivers: 80%), ‘support’ (21%/45%), and ‘patient anxiety’ (36%/28%). With regard to items rated as least affected (= 0), ‘information’ (84%/49%), ‘support’ (53%/31%), ‘life not worthwhile’ (17%/29%), and ‘not wasted time’ (99%/88%) showed a clinically relevant difference. These data show that SPC teams’ assessments are more favorable for most symptoms and concerns, but not for all. The full report of categorized proxy assessments is presented as Supplementary Material (see Supplement File 1, Table S2). We observed significant correlations between the assessments of SPC teams and family caregivers concerning ‘pain’ (*r*_*s*_ = 0.357, *p* = .006), ‘other symptoms’ (*r*_*s*_ = 0.271, *p* = .041) and ‘family anxiety’ (*r*_*s*_ = 0.314, *p* = .018) with small to moderate effects. For all other items, no significant correlation could be observed. Except fair agreement for ‘pain’ (*κ*_*w*_ = 0.259, p = .007), ‘other symptoms‘ (*κ*_*w*_ = 0.252, p = .013) and ‘family anxiety’ (*κ*_*w*_ = 0.218, p = .019), concordance of ratings ranged between poor and slight agreement.

**Table 3:** Agreement and correlation between proxy assessments of family caregivers and SPC teams (paired N = 60)

POS item		Patients (N)	Weighted Cohen’s Kappa (k_*w*_) for grouped scores (3 Categories)	Significance level (k_*w*_)	Interpretation (k_*w*_)^a^	Spearman’s correlation coefficient (r_s_) for grouped scores (3 Categories)	Significance level (r_s_)
1	Pain	58	0.259	.007**	Fair	0.357	.006**
2	Other symptoms	57	0.252	.013*	Fair	0.271	.041*
3	Patient anxiety	56	0.093	.347	Slight	0.179	.186
4	Family anxiety	57	0.218	.019*	Fair	0.314	.018*
5	Information	57	-0.098	.200	Poor	-0.185	.167
6	Support	57	-0.038	.709	Poor	-0.023	.863
7	Life worthwhile	57	0.065	.531	Slight	0.141	.300
8	Self-worth	56	0.122	.251	Slight	0.205	.144
9	Wasted time^b^	58	0.000		Slight	n.a.	n.a.
10	Personal affairs	48	0.114	.324	Slight	0.155	.293

^a^ as defined by Landis and Koch (1977) [30]; ^b^ due to missing variance (ceiling effects) in proxy assessments

Level of significance: * p < .05; ** p < .01

Abbreviations: POS, Palliative Care Outcome Scale; n.a., not applicable

### Factors associated to the proxy assessment of SPC teams

We analyzed the impact of patient-related demographic, disease- and care-related factors as well as the dying situation on SPC outcomes as reported by SPC teams (dependent variable: POS total score, with higher scores representing worse SPC outcomes). Findings of multiple linear regression analysis are reported in Table 4. As highest VIF was 1.91, we concluded that multicollinearity was not a problem in our study [35]. The regression model explained 28.8% of variance in levels of POS total scores and three predictive factors emerged. In order of magnitude these were: patient age, disease type and location at death. Patient age had the largest effect, with younger age significantly predicting worse SPC outcomes (*ß* = -0.262, *p* = .001). Dying from a malignant disease showed a significant advantage for SPC outcomes over non-malignant diseases (*ß* = -0.236, *p* = .003). Further, dying at home showed a significant advantage for SPC outcomes over not dying at home (*ß* = 0.243, p = .006). As estimated by the regression, a hypothetical older patient with a malignant disease who died at home could expect better SPC outcome as measured by the POS proxy-version for teams. According to Cohen’s classification of effect sizes, all effects are small [31].

Factors influencing family caregivers’ reports of SPC outcomes in the patient’s last days of life could not be analyzed due to the limited number of available assessments.

**Table 4:** Factors associated to the SPC teams’ reports of SPC outcomes (N = 142)

	Dependent variable: POS total score (0–40)		
**Independent variables**	B (95% CI)	Standardized Beta	p-value
Patient age^a^	-0.150 (-0.241; -0.060)	-0.262	0.001**
Patient gender (0 = female; 1 = male)	0.760 (-1.257; 2.776)	0.060	0.457
Type of disease (0 = malignant; 1 = non-malignant)	-5.260 (-8.743; -1.776)	-0.237	0.003**
Having children (0 = yes; 1 = no)	-2.400 (-4.996; 0.197)	-0.160	0.070
Living environment (0 = together or close with family; 1 = living alone)^a^	0.318 (-2.301; 2.936)	0.021	0.811
Advance directive^a^ (0 = yes; 1 = no)	1.988 (-0.135; 4.111)	0.146	0.066
Time between initiation of SPV and death	-0.020 (-0.043; 0.002)	-0.185	0.075
Number of transfers between SPC settings	0.528 (1.650; 0.524)	0.100	0.340
Location at death (0 = at home; 1 = not at home ^b^)	3.087 (0.914; 5.260)	0.243	0.006**
Constant	23.769 (16.931; 30.607)		< 0.001
R^2^	0.288		

^a^ at the time of initiation of SPC; ^b^ not at home: nursing home, inpatient hospice or palliative care ward

Level of significance: * p < .05; ** p < .01; *** p < .001

Abbreviations: CI, confidence interval; POS, Palliative Care Outcome Scale; SPC, specialist palliative care

### Problems solved ‘particularly well’ or ‘inadequate’ in the patient’s last days of life

Qualitative analysis of free-text answers and subsequent quantification revealed more ‘particularly well’ than ‘inadequately’ solved problems within the patients’ last 7 days of life. While ‘particularly well’ solved problems were reported by SPC teams in 112 of 142 cases (78.9%) and by family caregivers in 39 of 60 cases (65.0%), ‘inadequately’ solved problems were stated in 53 of 142 cases (37.3%) and 25 of 60 cases (41.7%), respectively (data not shown).

Main categories of ‘particularly well’ solved problems from the SPC team perspective (N = 112) were physical problems (at least one of such problem indicated in 54%), followed by care-related problems (18%), psychosocial problems (22%), and family caregiver-related problems. Family caregivers’ main categories of ‘particularly well’ solved problems (N = 39) related to physical problems (62%), psychosocial problems (28%), and care-related problems (18%).

Regarding ‘inadequately’ solved problems, main categories among SPC teams (N = 53) related to physical problems (at least one of such problem indicated in 36%), family caregiver-related problems (34%), care-related problems (21%), and psychosocial problems (19%). From the family caregiver perspective (N = 25), main categories of ‘inadequately’ solved problems were physical problems (64%), care-related problems (16%), psychosocial problems (12%) and family caregiver-related problems (12%).

Details on qualitative analyses including subcategories and illustrative quotes as well as quantification of (sub-)categories are presented in Table 5 for SPC teams and in Table 6 for family caregivers.

**Table 5:** Qualitative analysis of free-text answers from specialized palliative care teams on ‘particularly well’ and ‘inadequately’ solved problems

	‚Particularly well‘ solved problems (N = 112)			‚Inadequately‘ solved problems (N = 53)	
**Categories and Subcategories** ^**a**^	**n (%)** ^a^	**Illustrative quotes** ^b^		**n (%)** ^a^	**Illustrative quotes** ^b^
**Physical problems**	**61 (54.5)**			**19 (35.8)**	
Symptom control (general)	10 (8.9)	´Good symptom control at the end of life´			
Pain	23 (20.5)	´Pain relief by PCA pump´		4 (7.5)	´Pain treatment; severe pain due to decubitus ulcer´
Dyspnea	11 (9.8)	´Dyspnea control´		1 (1.9)	´Dyspnea´
Gastrointestinal symptoms	2 (1.8)	´Significant reduction of fecal vomiting due to insertion of a nasogastric tube´		4 (7.5)	´Patient’s distress due to massive vomiting could not be alleviated by invasive mechanical solutions (placement of a drain tube) because of the patient’s non-compliance´
Agitation/restlessness	7 (6.3)	´Restlessness´		5 (9.4)	´Pronounced terminal agitation two nights before death, on-call staff reacted inadequately´
Other physical issues	3 (2.7)	´Wound care´, ´Preparations taken for the occurrence of bleedings were very good´		3 (7.5)	´Heavy mucous secretion´
Palliative sedation	5 (4.5)	´Symptom relief by palliative sedation´		2 (3.8)	´No agreement between patient and team regarding medication and sedation’
**Psychosocial problems**	**25 (22.3)**			**10/53 (18.9)**	
Psychosocial care and emotional comfort	12 (10.7)	´Providing a sense of security´, ´Psychosocial support´		3 (5.7)	´The patient’s emotional reserve ´
Anxiety	6 (5.4)	´Attending to the patient’s future- and care-related fears´, ´Panic attacks due to breathlessness´		2 (3.8)	´Aiding the patient to be anxiety-free and relaxed´
Coping with the disease	4 (3.6)	´Patient’s acceptance of the situation´		4 (7.5)	´Patient denied disease-related discussions´
Autonomy/self-determination, wishes	6 (5.4)	´Autonomy was preserved´, ´Patient’s individual (care-related) needs and ideas were fully taken into account´		1 (1.9)	´Attention to patient perspective (wishes, experience) - limited communication because of time constraints’
**Care-related problems**	**32 (28.6)**			**11 (20.8)**	
Dying at home or at the desired place of death	23 (20.5)	´Home death was made possible´			
Care transitions	7 (6.3)	´Transfer to hospice prior to breakdown of the husband´		2 (3.8)	´Transfer to hospice did not take place´
Cooperation of involved services	4 (3.6)	´In cooperation with the nursing home staff, the patient received optimal care´		2 (3.8)	´No patient care attendant could be organized´
Terminal care	15 (13.4)	´Terminal care´		1 (1.9)	´Terminal phase recognized too late by part of the team´
General care issues				7 (13.2)	´No aids and appliances supplied, as the couple refused them´, ´Basic nursing – family caregivers approved a nursing service very late´
**Family caregiver-related problems**	**15 (13.4)**			**18 (34.0)**	
Family caregiver care and counselling	36 (32.1)	´Support of the wife at home´, ´Family system preserved, husband was able to care until the end´		11 (20.8)	´Contact with family caregivers and counselling´
Difficult family dynamics				8 (15.1)	´Family caregivers conceptions of care often divergent from those of the patient´

^a^ Multiple answers possible; n/% relate to having reported at least one problem in the respective (sub-)category; ^b^ Quotes are translations of original written responses of specialized palliative care teams to open-ended questions

Blank fields mean no mention in the free-text answers

**Table 6:** Qualitative analysis of free-text answers from family caregivers on ‘particularly well’ and ‘inadequately’ solved problems

	‚Particularly well‘ solved problems (N = 39)			‚Inadequately‘ solved problems (N = 25)	
**Categories and Subcategories** ^**a**^	**n (%)** ^a^	**Illustrative quotes** ^b^		**n (%)** ^a^	**Illustrative quotes** ^b^
**Physical problems**	**24 (61.5)**			**16 (64.0)**	
Pain	18 (46.2)	´Tumor pain well controlled’, ´Pain management´		2 (8.0)	´Pain´,´Pain management´
Dyspnea	2 (5.1)	´Shortness of breath´		1 (4.0)	´Shortness of breath´
Gastrointestinal symptoms	3 (7.7)	´Nausea´, ´Stomach cramps´		7 (28.0)	´Nausea could only be relieved a little´, ´Nausea, vomiting, constipation´
Agitation/restlessness				1 (4.0)	´His getting up at nights, out of the bed and jiggling everything […]!´
Dry mouth				3 (12.0)	´Dry mouth and lips´, ´Dry mouth´
Other physical issues	3 (7.7)	´Cough´		3 (12.0)	´Walking alone – showering´, ´Open back´
**Psychosocial problems**	**11 (28.2)**			**3 (12.0)**	
Psychosocial care and emotional comfort	7 (17.9)	´Caregiving was mindful and respectful´, ´Personal attention was always available´		1 (4.0)	´Emotional support, he was very unsettled by permanently changing palliative staff in the team.´
Anxiety	2 (5.1)	´Anxiety´, ´Addressing the patient’s fear that the family would have to take care of everything´			
Coping with the disease	3 (7.7)	´Clarify questions in peace´, ´Any personal questions regarding the disease´			
Autonomy/self-determination, wishes				2 (8.0)	´Ending her suffering. Her wish to die, as active euthanasia is not yet legal´
**Care-related problems**	**7 (17.9)**			**4 (16.0)**	
Dying at home or at the desired place of death	3 (7.7)	´That the patient was discharged home to his familiar environment´, ´That he was allowed home from the hospital at the end´		1 (4.0)	´No coming back to home [for dying]´
General care issues	4 (10.3)	´Medical care´, ´The medication scheduling´		3 (12.0)	´The patient could have been checked more often´, ´Physician visits much too late´
**Family caregiver-related problems**				**3 (12.0)**	
Family caregiver care and counselling				3 (12.0)	´Presence of us (confidants) when he died. We had been promised that we would be called when the time came. It was foreseeable on the morning of the day he died, but we were not informed until after his death.´

^a^ Multiple answers possible; n/% relate to having reported at least one problem in the respective (sub-)category; ^b^ Quotes are translations of original written responses of family caregivers to open-ended questions

Blank fields mean no mention in the free-text answers

## Discussion

Our study aimed to explore SPC outcomes in the patient’s last days of life from the perspectives of SPC teams and family caregivers. The study demonstrates the range and type of symptoms and other concerns reported in patients’ last days.

Overall, the reported SPC outcome, as reflected by the multidimensional POS total score, was slightly better in SPC teams than in family caregivers. Across both perspectives, POS items rated as least affected related to no waste of time, patient information and support in terms of patient’s ability to share feelings. However, satisfaction rates were consistently lower in family caregivers. Most strikingly, satisfaction with patient information was prevalent in two-thirds of SPC teams, but only in half of family caregivers. Although still one of the aspects rated as least affected, lower ratings may rather mirror family caregivers’ own dissatisfaction with the type, volume or timing of information in the last days of the patient’s life than dissatisfaction of the patient him- or herself. During SPC and as death approaches, family caregivers’ need for information remains high, but seems less met within the patient’s last 7 days of life compared to the beginning of inpatient SPC [36].

Items congruently rated as most affected by both groups related to patient anxiety, patients feeling regarding worthiness of life (‘life not worthwhile’), and patients feeling good about themselves (‘no self-worth’). Prevalence of such ratings were each higher in family caregivers than in SPC teams. These items reflect emotional aspects of end-of-life situations, and evidence points out that psychological symptoms and problems related to the patient’s well-being may be overestimated by family caregivers [17]. Caring for a loved one lasts until his/her death, which is a highly demanding and sometimes overwhelming situation for family caregivers [37]. Uncertainty or worries regarding the emotionality of the patient may affect family caregivers own psychological well-being. Thus, emotional support for family caregivers in terms of being listened to, cared for and empathized with, as well as compassionate communication about their estimates of the patient’s emotionality, are paramount.

Although based on a small-scale subgroup of 60 patients, our findings suggest that the correlation and agreement between proxy assessments of SPC teams and family caregivers was rather low, with family caregiver scores usually indicating poorer outcome. Correlational effects for problems related to pain, other symptoms and family anxiety were small to moderate; however, using weighted kappa (κ_w_) statistics, substantial agreement as defined by Landis and Koch [30] was not found in any of the given problems. This is consistent with the existing literature, mainly reporting discrepancies in family caregivers’ and health care professionals’ assessments of SPC outcome [19, 21, 38]. Empirical evidence indicates that agreement between family caregivers and health care professionals seems best for physical aspects of end-of-life care, especially for pain [19], while agreement for psychological aspects is low [21]. A study that also used the POS for comparing symptom assessment of nurses and family caregivers showed the latter to be more reliable proxies, but both groups tended to overestimate patient’s psychological symptoms [19]. A further study confirmed that nurses and family caregivers overestimated psychological, functional, and existential symptoms compared to patients’ self-reports, but underestimated physical and social symptoms [38]. Our findings hold several potential clinical impacts: Firstly, health care professionals should be aware of the low levels of agreement between professional and family caregiver respondents’ estimations of patients’ SPC outcomes and of how any misperceptions or differences in perspective might affect the family or patient. For example, differences might manifest in conflict when family caregivers are disappointed because they feel that the SPC team does not take the patient’s problems and needs seriously. Secondly, more communication about end-of-life issues between SPC teams and family caregivers should be encouraged, particularly with regard to psychological aspects. Beyond benefits of shared perspectives for the patient’s care and comfort during his/her last days of life, family caregivers’ perceptions of the patients dying are key for their own well-being and their grieving process [39, 40]. Lastly, notwithstanding, the patient’s self-assessment is imperative, but patient’s ability to report symptoms may diminish as illness progresses and the need for proxy responders sometimes arises. Nonetheless, a study on routinely collected PROMs in palliative care showed that in most clinical scenarios patient-provided self-reports are feasible [41]. Training and guidance for health care professionals in palliative care on how to implement and support patient- (and proxy-)reported outcomes could improve the use of PROMs [42]. If proxy-assessment is due, our findings – together with the existing evidence – highlight the need to consider both the family caregiver and the health care professional perspective when caring for a dying patient.

Further, we were interested in identifying factors associated with the reported SPC outcome in the patient’s last days of life, as reflected by the POS total score. While we waived respective analysis for family caregiver assessments due to the limited amount of data, multivariable regression analysis was feasible in SPC teams. We found younger patient age, a non-malignant disease and not having died at home to be predictors of worse SPC outcomes. In line with our results, studies repeatedly report dying at home to predict better quality of death and dying [9, 43]. With regard to patients who had died from a non-malignant disease, evidence shows that patients are less likely to be aware of their prognosis, are more likely to have unmet palliative care needs, and access SPC services often late in the disease trajectory [44–46]. These aspects may add complexity to SPC for patients with non-malignant diseases and may negatively affect SPC outcomes in the patient’s last days of life as reported by SPC teams. As for the impact of age, it is well known that care delivery for younger people dying from incurable illnesses imposes a specific burden to health care professionals [47], which may shape SPC teams’ critical reports on SPC outcomes in the patient’s last days of life. However, more research is warranted to gain a better understanding on the relationship of SPC outcome as assessed by SPC teams and patient age.

Navigating the dying days is challenging to both SPC teams and family caregivers. However, our analysis of problems solved ‘particularly well’ shows that SPC seems to address the main problems patients face in their last days of life. Problems solved ‘inadequately’ were less often reported, but covered the full range of challenging problems in end-of-life care. Particularly, written accounts point out room for improvement regarding family caregiver care and the psychosocial and spiritual dimension of palliative care in the patient’s last days of life. This is in line with a previous study, which also identified insufficient professional support for informal caregivers and inadequate psychosocial support for patients as unsolved problems [48]. Our findings underline the unique, complex and multilayered situation when life ends, as problems of similar nature were encountered as problems solved ‘particularly well’ as well as ‘inadequately’.

### Limitations

The relatively small numbers of family caregivers, who completed the assessment post-bereavement, limits our study. Unfortunately, we lack reliable knowledge about the proportion of potentially eligible family caregivers among the 142 studied patients. Thus, we cannot estimate non-responder analyses and can only report on a convenience sample. Further, over 90% of participants presented with an oncological disease. In Germany, the majority of patients in SPC services are cancer patients [49]. Yet, given that SPC’s embracing approach includes all people with a life-limiting disease, this can be another limitation. Lastly, the examination of agreement between SPC teams and family caregivers based on categorized POS items scores [6]. That is, the most positive answer was left in its own category, while the other response options were collapsed into two new categories. A different categorization scheme, leaving the midpoint in its own category, might have resulted in more balanced categories.

## Conclusion

The study demonstrates the range of symptoms and other concerns occurring in the patient’s last days. Overall, care seems to be perceived as adequate until the very end of patients’ lives according to proxy assessments. However, our study revealed that improvements in family caregiver care and psychosocial and spiritual issues may benefit high quality SPC until the patient’s death. Proxy measures should be implemented when the patient’s health deteriorates to ensure systematic assessment of problems and concerns. Close communication between family caregivers and SPC teams is paramount during the patient’s last days of life in order to address his/her needs in the best possible way, as agreement by proxies was low for most symptoms and problems. Reasons for low agreement of health care professionals and family caregivers deserve further research and awareness in daily clinical practice. Further, future research should focus on factors related to family caregivers’ reports of SPC outcomes in the patient’s last days of life and compare those to factors associated with the reports of SPC teams.

### Electronic supplementary material

Below is the link to the electronic supplementary material.

Supplementary Material 1

## Data Availability

The datasets generated and/or analyzed during the current study are not publicly available, but are available from the corresponding author (AU) on reasonable request and with authorization of the data protection officer of the University Medical Center Hamburg-Eppendorf, Hamburg, Germany.

## Abbreviations

CODE™: Care of the Dying Evaluation
ECOG: Eastern Cooperative Oncology Group
HOPE: Hospice and Palliative Care Evaluation
IPOS: Integrated Palliative Care Outcome Scale
POS: Palliative Care Outcome Scale
PROMS: Patient reported outcome measures
QoDD: Quality of Dying and Death Questionnaire
SPC: Specialist palliative care
TI: Tolerance indices
VIF: Variance inflation factor
