# Supplementary material for: Specialist palliative care until the very end of life - reports of family caregivers and the multiprofessional team
Source: BMC Palliat Care. 2023 Oct 10;22:153. doi: 10.1186/s12904-023-01266-6 (PMC10563273; doi:10.1186/s12904-023-01266-6)
Supplement: Supplementary file 1 — Supplementary Material 1 [file 12904_2023_1266_MOESM1_ESM.docx]

**Supplement File 1**

**Specialist palliative care until the very end of life - reports of family caregivers and the multiprofessional team**

**Table S1** Mean proxy assessments of family caregivers and SPC teams: POS items and total score (paired N=60)

| POS item ^a^  Over the last three days of the patient’s life: | | N | SPC Team  M (SD) | Family caregivers  M (SD) | Significance level  (paired t-test) |
| --- | --- | --- | --- | --- | --- |
| 1 | Pain | 58 | 1.5 (1.2) | 1.8 (1.2) | .176 |
| 2 | Other symptoms | 57 | 2.2 (1.1) | 2.0 (1.4) | .226 |
| 3 | Patient anxiety | 56 | 1.8 (1.4) | 1.6 (1.3) | .878 |
| 4 | Family anxiety | 57 | 2.4 (1.4) | 3.1 (1.2) | .001** |
| 5 | Information | 57 | 0.2 (0.7) | 0.9 (1.1) | .001** |
| 6 | Support | 57 | 1.2 (1.5) | 2.0 (1.7) | .010* |
| 7 | Life worthwhile | 56 | 2.3 (1.5) | 2.0 (1.7) | .277 |
| 8 | Self-worth | 52 | 2.1 (1.5) | 2.0 (1.5) | .825 |
| 9 | Wasted time | 58 | 0.0 (0.0) | 0.3 (1.0) | n.a. ^b^ |
| 10 | Personal affairs | 48 | 0.6 (1.1) | 0.9 (1.4) | .173 |
| POS total score (scale: 0-40) ^c^ | | 56 | 14.2 (6.6) | 16.6 (6.9) | .039* |

^a^ Questions 1 to 8: range 0 (no problems) to 4 (overwhelming problems, questions 9 and 10: scores of 0, 2, or 4 (higher scores indicate more problems); ^b^ due to missing variance (ceiling effects) in proxy assessments; ^c^ lower scores indicate better SPC outcome

Level of significance: * p<.05; ** p<.01

Abbreviations: POS, Palliative Care Outcome Scale; SPC, specialist palliative care

**Table S2** Categorized proxy assessments of family caregivers and SPC teams: POS items (paired N=60)

| POS item ^a^  Over the last three days of the patient’s life: | |  | 0  (% rated as least affected) | 1-2  (%) | 3-4  (% rated as most affected) |
| --- | --- | --- | --- | --- | --- |
| 1 | Pain | SPC Team | 27.5 | 45.1 | 27.5 |
|  |  | Family caregivers | 22.4 | 41.4 | 36.2 |
| 2 | Other symptoms | SPC Team | 9.9 | 51.1 | 39.0 |
|  |  | Family caregivers | 17.2 | 41.4 | 41.4 |
| 3 | Patient anxiety | SPC Team | 28.3 | 35.5 | 36.2 |
|  |  | Family caregivers | 21.1 | 50.9 | 28.1 |
| 4 | Family anxiety | SPC Team | 8.7 | 38.4 | 52.9 |
|  |  | Family caregivers | 3.4 | 16.9 | 79.7 |
| 5 | Information | SPC Team | 83.7 | 14.2 | 2.1 |
|  |  | Family caregivers | 49.1 | 42.1 | 8.8 |
| 6 | Support | SPC Team | 52.6 | 26.7 | 20.7 |
|  |  | Family caregivers | 31.0 | 24.1 | 44.8 |
| 7 | Life worthwhile | SPC Team | 17.0 | 40.7 | 42.2 |
|  |  | Family caregivers | 29.3 | 27.6 | 43.1 |
| 8 | Self-worth | SPC Team | 18.3 | 45 | 36.6 |
|  |  | Family caregivers | 15.8 | 45.6 | 38.6 |
| 9 | Wasted time | SPC Team | 99.3 | 0.7 | 0 |
|  |  | Family caregivers | 87.9 | 6.9 | 5.2 |
| 10 | Personal affairs | SPC Team | 73.1 | 19.0 | 7.9 |
|  |  | Family caregivers | 66.7 | 22.8 | 10.5 |

^a^ Questions 1 to 8: range 0 (no problems) to 4 (overwhelming problems), questions 9 and 10: scores of 0, 2, or 4 (higher scores indicate more problems)

Abbreviations: POS, Palliative Care Outcome Scale; SPC, specialist palliative care
